# Combined central retinal vein and cilioretinal artery occlusion in a 25-year-old woman

**DOI:** 10.22336/rjo.2022.35

**Published:** 2022

**Authors:** Lech Sedlak, Marta Świerczyńska, Dorota Pojda-Wilczek

**Affiliations:** * Department of Ophthalmology, Faculty of Medical Sciences in Katowice, Medical University of Silesia, Katowice, Poland; ** Department of Ophthalmology, Kornel Gibiński University Clinical Center, Medical University of Silesia, Katowice, Poland

**Keywords:** central retinal vein occlusion, CRVO, cilioretinal artery occlusion, CLRAO, methylenetetrahydrofolate reductase gene polymorphism, MTHFR polymorphism, A1298C mutation

## Abstract

We report a case of a 25-year-old woman with sudden and painless diminution in vision and central scotoma in her left eye (LE). She was a smoker and had been taking combined oral contraceptive (COC) pills for 1 year. On admission, the best-corrected visual acuity (BCVA) was 1,5/50 in the LE.

Posterior segment examination revealed optic disc edema with flame-shaped retinal hemorrhages, mildly tortuous and dilated retinal veins. Moreover, retinal edema in the peripapillary and perimacular region, foci of hemorrhages and Roth’s spots in the posterior pole, as well as pale superior papillomacular bundle were observed.

Fundus fluorescein angiography (FFA) confirmed the delayed flow of contrast through the cilioretinal artery in the LE. The clinical picture suggested left central retinal vein (CRVO) with cilioretinal artery occlusion (CLRAO). All laboratory and imaging tests were normal except for homozygous methylenetetrahydrofolate reductase (MTHFR) gene mutation (A1298C genotypes). However, serum homocysteine (Hcy) level was normal. Low molecular weight heparin (LMWH) treatment was administered. Retinal lesions, as well as BCVA improved, but central scotoma remained.

**Abbreviations:** aPTT = activated partial thromboplastin time, BCVA = best-corrected visual acuity, CBC = complete blood count, CLRAO = cilioretinal artery occlusion, COC = combined oral contraceptive, CRA = central retinal artery, CRP = serum C-reactive protein, CRVO = central retinal vein occlusion, CT = computed tomography, CTA = computed tomography angiography, ECG = electrocardiography, ESR = erythrocyte sedimentation rate, FERG = flash electroretinogram, FFA = fundus fluorescein angiography, GCA = ganglion cell analysis, GCL = ganglion cell layer, Hcy = homocysteine, ICGA = indocyanine green angiography, INR = international normalized ratio, IOP = intraocular pressure, IPL = inner plexiform layer, LE = left eye, LMWH = low molecular weight heparin, mfERG = multifocal electroretinogram, MTHFR = methylenetetrahydrofolate reductase, OCT = optical coherence tomography, RE = right eye, VF = visual field

## Introduction

Combined central retinal vein occlusion (CRVO) and cilioretinal artery occlusion (CLRAO) is a rare variant of retinal vascular disease, which causes sudden unilateral visual acuity loss [**1**]. Combined CRVO and CLRAO comprises 27% to 62% of all CLRAOs [**1**,**2**]. Moreover, non-arteritic CLRAO alone and arteritic CLRAO associated with giant cell arteritis or with ischemic optic neuropathy may be reported [**3**].

The most important risk factors for CRVO (and combined CRVO/ CLRAO) are the same as those for atherosclerosis (advanced age, hypertension, hyperlipidemia, diabetes, cigarette smoking, positive family history). Other systemic predisposing factors include inherited and acquired thrombophilia, high blood viscosity, systemic vasculitis, and autoimmune disease [**4**-**6**]. The most common hematologic abnormalities among young individuals with CRVO are hyperhomocysteinemia and antiphospholipid antibodies [**6**].

## Case presentation

A 25-year-old woman presented to the emergency department with sudden and painless decreased visual acuity and central scotoma in her left eye (LE) that began 6 hours earlier. The patient also reported an episode of sudden blurring of vision in her LE that occurred 7 days before and lasted about 2 hours with spontaneous full recovery. She had no history of infectious diseases, trauma, or other systemic complaints. She had no cardiovascular risk factors except for cigarette smoking (15 cigarettes daily) for 5 years and using a combined oral contraceptive (COC) pill (ethinyl estradiol 0.03 mg and drospirenone 3 mg) for 1 year. 

**Fig. 1:**
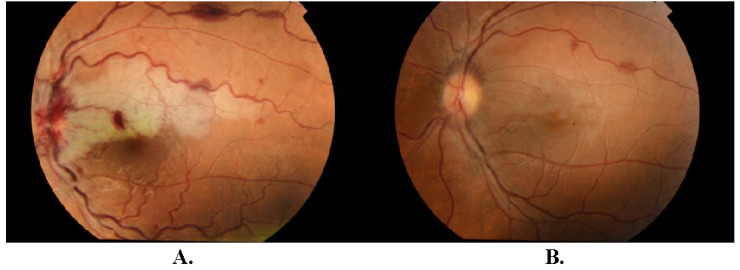
Fundus photographs of the LE. **A.** Day post presentation: an optic disc edema with flame-shaped retinal hemorrhages, mildly tortuous and dilated retinal veins, retinal edema in the peripapillary and perimacular region, foci of hemorrhages and Roth’s spots in the posterior pole and pale superior papillomacular bundle. **B.** 3 months post presentation: the hemorrhages were absorbed; the dilatation and tortuosity of retinal veins subsided.

At the time of presentation, the best-corrected visual acuity (BCVA) was 5/5 in the right eye (RE) and 1,5/50 in the LE. The anterior segment evaluation was within the normal range except for mild relative afferent pupillary defect of the LE. A left fundus examination revealed blurred optic disc margin with flame-shaped retinal hemorrhages, mildly tortuous and dilated retinal veins. Moreover, retinal edema in the peripapillary and perimacular region, foci of hemorrhages, Roth’s spots in the posterior pole and pale superior papillomacular bundle were observed (**Fig. 1 A**). No abnormalities were detected in the picture of the RE fundus. Intraocular pressure (IOP) was 12 mmHg in the RE and 14 mmHg in the LE.

An urgent neurological consultation showed no focal central nervous system signs. Her body mass index, blood pressure, heart rate, electrocardiography (ECG) was normal. Laboratory workup including the complete blood count (CBC), erythrocyte sedimentation rate (ESR), serum C-reactive protein (CRP), random blood sugar level, renal function tests, activated partial thromboplastin time (aPTT), international normalized ratio (INR), extended lipid profile, D-dimer were within normal limits. Results of B-hCG pregnancy testing were negative. The head computed tomography (CT) and computed tomography angiography (CTA) of the cerebral arteries were unremarkable. The patient was referred for further ophthalmic treatment, but she did not consent to hospitalization.

**Fig. 2:**
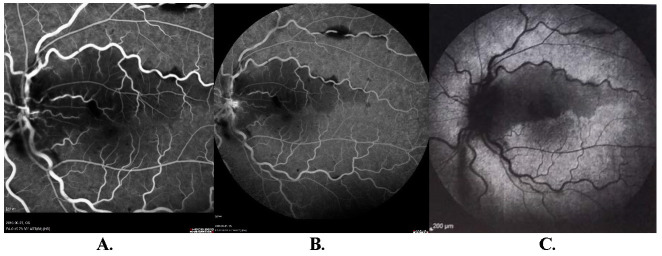
**A, B.** AFF of the LE 1-day post presentation: delayed contrast flow in the cilioretinal artery, well-maintained flow in the main retinal vessels, hypofluorescent areas from the foveola to the upper vascular arc, the florescence blockage in the projection of scattered intraretinal hemorrhages, the contrast stasis on the optic disc in the late phase of the angiogram. **C.** ICGA of the LE 1-week post presentation: maintained flow thorough the choriocapillaris and retinal vessels, the blockage in the projection of the hemorrhages, Extensive area of blockage below the upper temporal vascular arch from the optic disc through the upper part of the macula to the temporal equator.

The woman came the next day to the Ophthalmology Clinic, her BCVA was 0,5/50 in the LE, and then was admitted to the Department of Ophthalmology for further work-up. Low molecular weight heparin (LMWH) treatment was administered. In fundus fluorescein angiography (FFA), the delayed flow of contrast through the cilioretinal artery in the LE was described. Moreover, an area of hypofluorescence extending from the fovea to the upper vascular arch in the posterior pole, fluorescence blockage in the projection of diffuse intraretinal hemorrhages, dilatation, and tortuosity of the venous vessels, and in the late phase, contrast stasis on the optic nerve disc were demonstrated. This clinical picture suggested left central retinal vein (CRVO) with cilioretinal artery occlusion (CLRAO) (**Fig. 2 A, B**).

**Fig. 3:**
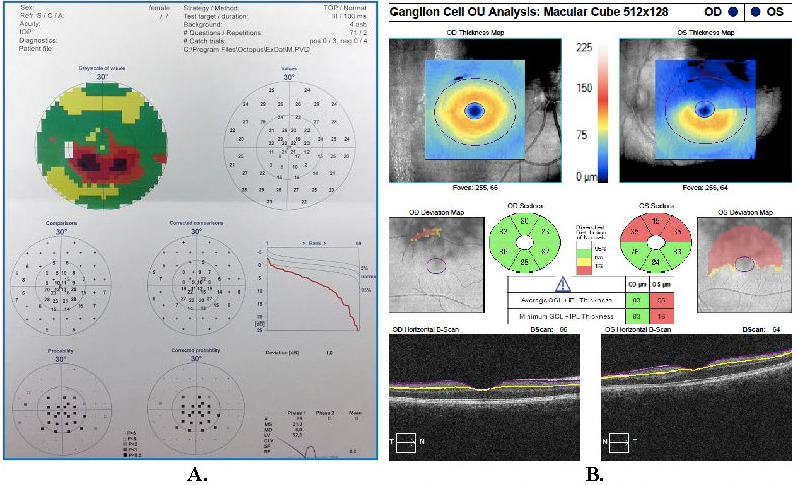
**A.** VF test of the LE 1-day post presentation. Central 24-2 threshold test performed with stimulus size III and SITA-FAST algorithm: numerical sensitivity and pattern deviation plots showing central scotoma. **B.** OCT – GCA of the RE: normal, and the LE: ganglion cell loss in the upper sectors.

**Fig. 4:**
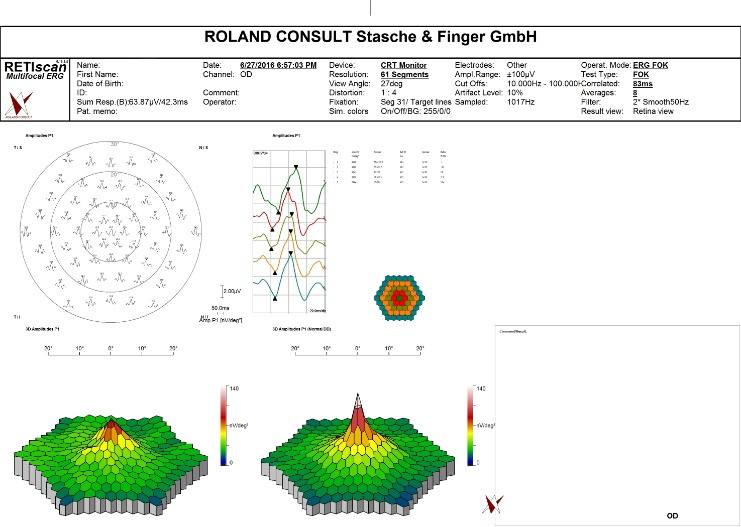
Multifocal electroretinogram (mfERG) analysis using a 103-hexagon stimulus display. Plotted waveform (top) and pseudocolor response density plots (bottom) of the LE. Lower response amplitudes in the central part compared to the RE. Responses in the central visual field correspond to the retinal region of visual loss in the LE.

Static visual field (VF) testing revealed the presence of a central scotoma in the LE (**Fig. 3 A**). Flash electroretinogram (FERG) was normal in both eyes. Multifocal electroretinogram (mfERG) of the LE showed lower amplitudes in the central part (**Fig. 4**), demonstrating a functional defect in the retinal area corresponding to the visual field defect. 

Despite the significant reduction of visual acuity on admission, after 4 days of hospitalization, BCVA of the LE was 5/6 looking slightly sideways. The patient was discharged with the recommendation of cardiological, rheumatological and hematological consultation for further diagnosis. 

On the 7th day of follow-up, indocyanine green angiography (ICGA) was performed. In the LE, zones of fluorescence blockage at the site of hemorrhages were visualized in the area of the optic nerve disc, macula and in the area of the upper vascular arch. Below the upper temporal vascular arch, a large area of fluorescence blockage from the optic nerve disc was described, reaching the upper part of the macula, and the vicinity of the equator in the temporal part, in the projection of the nerve fiber layer edema. Venous dilatation suggested obstructed venous outflow. The resulting image was uncharacteristic (**Fig. 2 C**). Hematological consultation was again recommended due to an image that might have suggested changes in the white blood cell or the coagulation system.

**Fig. 5:**
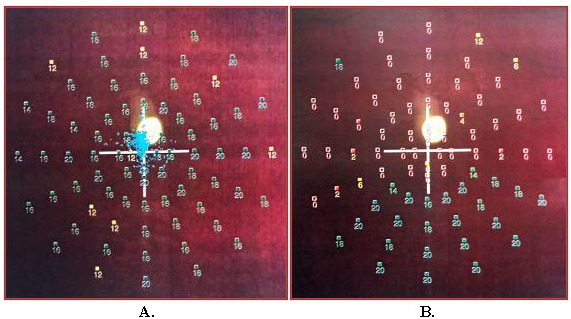
Microperimetry 3 months post presentation. **A.** The RE: normal. **B.** The LE: persisting defect in the central visual field

On the 21st day of follow-up, the patient was in a stable condition with satisfactory BCVA of 5/5 in the LE. The hemorrhages were absorbed, the dilatation and tortuosity of retinal veins subsided (**Fig. 1 B**), but the woman continued to report the presence of the central scotoma, as confirmed by the VF, microperimetry and mfERG results (**Fig. 5 A, B**). Ganglion cell analysis (GCA) using optical coherence tomography (OCT) showed that in the LE average ganglion cell layer (GCL) + inner plexiform layer (IPL) thickness was 55 μm and the minimum GCL and IPL thickness was 16 μm, whereas in the RE results were within normal limits (**Fig. 3 B**). 

The MRI of the orbits was normal. Tests for autoimmune disorders that could be the cause of vasculitis were negative. Detailed hematological diagnostics aimed at excluding conditions that lead to blood hypercoagulability showed no significant deviations from the norm. Genetic testing revealed the presence of A1298C subtype homozygous mutation for the gene encoding methylenetetrahydrofolate reductase (MTHFR), a vital enzyme in homocysteine (Hcy) metabolism. However, serum Hcy level was 11 μmol/ L (the normal range: 4-15 μmol/ L). The patient was also consulted by a cardiologist, dermatologist, and endocrinologist, who did not reveal any etiological factor. The woman stopped using COC pills and ceased smoking. At the 4-year clinical follow-up, no further thrombotic episodes were noted.

## Discussion

The pathomechanism of combined CRVO/ CLRAO still remains unclear. It is usually suggested that this phenomenon should be interpreted as a hemodynamic block. The perfusion pressure in the choroidal vascular bed is normally lower than in the central retinal artery (CRA) by approximately 7 mmHg [**1**]. During obstacle in the venous system, a rise in the intraluminal pressure from continuous blood pumping from the CRA is observed. Due to the choroidal origin, no autoregulatory mechanism to maintain blood flow exists in the CLRA [**7**]. 

In the case described, the presence of A1298C subtype homozygous mutation for the gene encoding MTHFR, a Hcy metabolic regulatory enzyme, which supports its conversion to methionine, was detected. The most recognized MTHFR gene polymorphism – the C677T mutation decreased the activity of the enzyme, which led to impaired Hcy methylation and further elevated plasma Hcy level. The A12988C MTHFR mutation in the homozygous or heterozygous state also reduces the activity of the enzyme in vitro, however, the Hcy level often remains normal [**8**], as in the case described. 

Hyperhomocysteinemia contributes to vascular damage by inhibiting endothelial cell growth and post-injury endothelial repair. Moreover, it redounds to vascular smooth muscle proliferation, inflammatory monocyte differentiation, oxidation of low-density lipoprotein and prothrombotic state [**8**,**9**]. For this reason, elevated Hcy concentration has been established as a risk factor for myocardial infarction, cerebrovascular events and one of the most common causes of RAO among young adults [**6**]. However, the relationship between MTHFR gene polymorphism and the risk of thromboembolic disease remains uncertain [**8**-**11**]. Gao et al. [**9**] indicate that MTHFR C677T polymorphism may increase the susceptibility to thromboembolism events in the Asian, but not in the Caucasian. Moreover, the authors could not confirm a significant association of MTHFR A1298C polymorphism and thromboembolism. 

In the reported case, the patient declared to have used COCs, which are a well-known risk factor for cardiovascular and cerebrovascular adverse events. Their use increases the blood coagulability, induces antifibrinolytic activity and may contribute to the change of the normal arterial wall architecture [**12**-**14**]. Moreover, smoking, also reported by the patient, is not only an important independent risk factor for thromboembolic phenomenon, but also increases the risk of clot formation in arteries in OCs users, possibly secondary to an increase in plasma fibrinogen, Factor VII, prothrombin, Factor XI, and Factor X concentrations. Smoking is reported to cause increased intravascular fibrin deposition, enhanced monocyte tissue factor expression and increased activation of intrinsic coagulation proteins [**12**,**15**,**16**]. Vascular occlusion due to the long-term use of OCs most often affected women in the older age groups [**16**]. However, there are reports of young women in whom, despite the lack of traditional risk factors for thromboembolic events, relatively short drospirenone-ethinyl estradiol use and smoking led to thrombus formation and retinal vessel occlusion, thus leading to severe clinical condition [**12**,**13**].

There are some controversies regarding antithrombotic therapy for CRVO. No benefit from treatment with antiplatelets or anticoagulants was observed. Moreover, a significantly greater severity of retinal hemorrhages was reported among aspirin users than among nonusers [**17**]. According to the guidance for the management of acute phase treatment of venous thrombosis, LMWH may be considered for the acute phase treatment of RVO. Further long-term treatment with acetylsalicylic acid should be based on individual indications for primary or secondary prevention of cardiovascular disease. It is essential that young patients with the combined occlusion of retinal artery and vein should be evaluated in detail because the differentiation between thrombotic and inflammatory etiology is crucial for proper management [**17**,**18**].

The prognosis for combined CRVO/ CLRAO is generally good. During follow-up, VA as well as visual field improvement is common in the non-ischemic CRVO/ CLRAO group. However, the involvement of the macula results in a persisting central scotoma even after the resolution of the venous stasis [**1**,**3**].

## Conclusions

Retinal vascular occlusions are relatively uncommon among young people and require thorough investigation to diagnose underlying conditions. Combined cases are often related to rheological causes like thrombophilia, vasculitis, and mechanical compression. However, the classic, often modifiable risk factors should not be forgotten. Timely diagnosis and quick initiation of appropriate management may help to restore visual function and prevent later possible life-threatening systemic complications. 

**Conflict of Interest**

Authors state no conflict of interest.

**Informed Consent and Human and Animal Rights statements**

Informed consent has been obtained from all individuals included in this study. 

**Authorization for the use of human subjects**

Ethical approval: The research related to human use complies with all the relevant national regulations, institutional policies, is in accordance with the tenets of the Helsinki Declaration and has been approved by the institutional review board of the Ophthalmology Department, Kornel Gibiński University Clinical Center, Medical University of Silesia, Katowice, Poland.

**Acknowledgements**

None. 

**Sources of Funding**

None. 

**Disclosures**

None.
